# The Occasional Serious Results of Pulp Treatment

**Published:** 1886-05

**Authors:** F. M. Dixon


AMERICAN JOURNAL

OF

DENTAL SCIENCE.

VOL. XX. THIRD SERIES.—MAY, 1886. No. 1.

ARTICLE I.

### THE OCCASIONAL SERIOUS RESULTS OF PULP TREATMENT.

BY F. M. DIXON, D. D. S.

[Read before the Odontological Society of Pennsylvania, February 6th.]

The discussion at a former meeting reminded me of a long contemplated intention, of writing a history of some of the more remarkable experiences of my professional life in the results of pulp extirpation. Believing that in such history there would be found both entertainment and material for profitable discussion, the paper having been written, is now offered for your consideration. It may be remembered, that during the discussion alluded to, it was remarked that the incidental results of root filling were frequently much more serious than most of us apprehended. Let us now see if the correctness of this position may not be amply proven by testimony which the following simple statement of facts will reveal. Not that imminent danger must necessarily attend at all times, even the most intricate operation needed upon a devitalized tooth, but that such results incident upon either present or subsequent circumstances, do frequently follow root-filling. Such circumstances for instance, as removal of the patient after a recently filled root, still in pathological conditions, out of reach of proper treatment, in case of periosteal inflammation ; or falling into the hands of a physician, who, however eminent or successful in general practice, may know positively nothing of the duties he assumes when undertaking those of a speciality which he has never even tried to comprehend.

The first case referred to was that of a young man for whom in the summer of 1854 I treated and removed the pulp of a superior lateral incisor, filling with gold, as was the practice of the time, as nearly as possible to the apical foramen. There was nothing apparent at its completion signifying that trouble was to be apprehended. It was with much surprise, therefore, that upon meeting the patient some days afterwards, I learned that he had been, and was still suffering from the effects of periosteal inflammation, with every indication that an abscess was forming. I advised him, as was I believe the universal practice at the time by those who undertook this kind of operating at all, to apply active fomentations with a view to hastening its termination as rapidly as possible, desiring him to report to me within the next twenty-four hours. Not hearing from him however for a number of days, and supposing that the crisis had passed, I was startled upon hearing that he was ill and under medical treatment. As I was about leaving the city, and the patient being a personal acquaintance, I called the next morning to make inquiry and was much astonished upon learning that during the night he had died of erysipelas of the stomach, as was reported by the physician in attendance. Although it was never supposed that death was caused by the treatment of the tooth of which I had every assurance by the family at the time, and again very recently, by a brother of the patient who was with him during his illness, yet the question has frequently arisen in my own mind as to whether it was not incidently even the cause of death. In order to reach a fair standpoint from which to judge, let us review the whole train of circumstances as they occurred after the fomentations had been recommended, which advice was not followed, but instead the physician's treatment, of which we have no knowledge, but do know of the agonizing pains frequently experienced during the process of suppuration.

We may naturally suppose that in this case they were exceptionally severe; that the patient's strength was greatly reduced by the rapidly developing but unsuspecting malady; while all the trouble was attributed to the condition of the tooth. At this period the physician was called in; it may have been at the very turning-point when remedial agencies must have proven unavailing, or even to him, there may have been no suspicion of the coming disease. Here then is illustrated the means by which the filled root may have been incident to the disaster; for during all this time the smouldering, unseen fires within were gaining strength, ere while to manifest the fearful ravages, which, unresisted, had been consuming the very vitals and had already gained that force which no human agency could control. Let us remember that erysipelas is but one of many diseases which need to be recognized in their earliest stages and combatted by proper remedies if danger or even death are to be arrested; and also that many thousands of pulpless roots annually filled, many of which remain from a few hours to a number of years so filled, ere manifesting such conditions as might produce results corresponding to those of the cited case. Though the fatal result in this instance might not have been arrested had the patient continued for three days under the dentist's treatment, yet this advantage would have been gained; that he would have known familiarity with the effects of forming abscesses, that the rapidly diminishing strength of the patient was due to something fiercer and stronger than this; and that the insidious enemy, whatever it was, must be immediately deprived of its formidable ally, even by removal of the tooth if needs be, and brought at once face to face with the skill that would have immediately discovered its nature and strength and administered timely remedies which, under God, might have worked its overthrow and saved the life of the patient.

He must be a dullard indeed, who would not profit by the experiences of such a case; as for myself, I have since approached with timidity the treatment of a threatened abscess, never omitting any possible precaution, by which it might be averted, especially where there are the slightest indications of the presence of either chronic or acute disease, which alone might prove to be all that the patient had strength to endure. A few such precautionary measures may be properly admitted here. The first of these was adopted about a year later.

The patient, an invalid, was waited on at her residence. Among the operations performed for her at the time, was one upon a bicuspid, of which the pulp was destroyed and removed and a temporary filling inserted. A few days later the tooth, seeming entirely well, was filled with gold, and for a time promised the best results. Nevertheless, about ten days later violent pains commenced, with other indications of acute periostitis. Upon visiting the patient I found her much prostrated. Whereupon the tooth was extracted and immediately returned to its place. Three days after which, with little or no suffering in the interim, it was perfectly comfortable, remaining so, I believe, until the day of her death, which occurred about twenty-five years later. We may now suppose a possible train of circumstances such as frequently do follow a series of operations resembling essentially those just described, less the extraction and replanting of the tooth, which would have been as likely to produce death, as those which followed the case first described. Supposing that this lady, already physically debilitated by protracted disease, had been visiting at Pottsville, where the case occurred, for medical and dental treatment, from a distant region where skilled medical or dental care could not be obtained. Anxious to return to family and friends, she had hurried off immediately upon completion of the last operation, and before the inflammatory conditions appeared. Now out of reach of intelligent treatment, still weak and debilitated from long continued sickness, who, for an instant, will doubt that the added torture of a growing abscess, and possibly blood poisoning, might have shortened her life by the quarter of a century that followed.

In 1862 I was visited by the late B. V. M., merchant of this city, who was suffering from the effects of acute pulpitis of the right inferior third molar. An application of the usual arsenical preparation was made, but in half an hour he returned, suffering so intensely and manifesting symptoms so unusual, that the tooth was unhesitatingly extracted. But being found with a perfectly healthy periosteum, was, by his consent, immediately returned to its socket. Whereupon the patient left with the understanding that he was to return to have it filled as soon after the soreness ceased as convenient, but did not reappear for nearly two months; then stated that at the time of the extraction of the tooth he was actually suffering from insipient malignant typhoid fever, from which he had only just recovered. Does it not seem more than probable that the pain, experienced just at this time, in the pulp, which had doubtless been exposed for a number of months, was caused by the approaching fever? And does it not seem also more than possible, that any considerable amount of pain added to what the patient was already necessarily subjected, may have proven just so much more than he was able to live through. And is it not reasonable to suppose that, in view of the existence of many thousands of half dead or dying, capped pulps, and more thousands of fragments left in obscure canal-ends; left to rot and fester upon provocation, will, or do, so act at periods just as critical as those described in the cases under consideration.

But in order to reach the exact animus of this question, in short let us do a little ciphering. We will suppose that in the United States alone there are in round numbers twelve thousand practicing dentists. Then consider the minimum of cases likely to lead to serious periostitis and acute alveolar abscess, if not properly treated just at the proper time, as two, to the most skillful operator in the profession, while to the least skillful we may safely attribute the maximum as fourteen per annum. This would average seven such cases per annum, which would aggregate 48,000 patients, who, under the present system of pulp treatment and root-filling, are subjected to the more than possible tortures and quite possible dangers arising from alveolar abscess incident thereto. Add to these sixteen per cent. as the most unsuccessful cases of nerve-capping, which will lead to similar results, and we have in round numbers 100,-000. This may be safely looked upon as the minimum of cases so treated annually.

A case interesting in this connection but resulting differently, was that of a young married lady who, some three years since, sent a request that I should visit her—as she was feeling too poorly to visit my office—for the purpose of treating a tooth, the roots of which I had filled ten years earlier, and which was now causing her great pain. Calling accordingly, I found her suffering intensely from inflammation of the parts, indicating unmistakably an approaching abscess, and as she expressed it, almost sick, which condition was attributed solely to the condition of the tooth. It required little skill, however, to discover that she was even then suffering from other causes, and that the then condition of the tooth was one of the results of that cause, all manifested by a generally depressed condition of the whole physical system. Believing it improper that, under the circumstances, she should be subjected to an hour's unnecessary suffering in addition to what seemed to be inevitably approaching from a cause, the nature of which was very certain, I immediately advised the removal of the tooth, and that her physician should be called in, remarking that, much as I valued the tooth, yet the general health being of much greater importance, we must now turn our special attention to that. She replied, "I see you think I am going to be sick, I will be guided by your advice," upon which her husband immediately procured a carriage and took her to have it removed. Upon returning home after the operation she went immediately to her bed, to which she was confined during the longest and severest illness of her life, finally, however, recovering. It is true she might have done so, even with the additional suffering to which she would have been subjected had the tooth been retained.

But who can tell? Certainly, no one. Yet these cases point sharply to the great care that should be observed ere undertaking the treatment of an incipient tumor of this kind, which may cause many hours of most intense suffering, lest, adding this to the debilitating effects of some possibly approaching deadly malady, it be made the incidental if not primary cause of death. And do they not point as clearly to a possibility that the M. D., and the D. D. S., each duly deferring to the other's skill in his specialty, may frequently work together to much greater advantage than when either undertakes the duties of the other, or of both? And if so, it is no less true because in their united judgment they do not always correctly diagnos or succeed in warding off the more serious effects of a failure to comprehend the true nature of a lesion or its cause. And that this is not mere corollary, let us consider the testimony of the following cases and profit by their many suggestions.

About fifteen years since, a young but very intelligent physician brought for consultation a gentleman who had been suffering for about three years with an abscess of the inferior maxilla, which had during that period been discharging into a heavy beard from a fistulous opening immediately under the angle of the right side. Its history was stated substantially as follows: Three years prior to this time there had been filled with gold a large cavity in the distal surface of the right inferior second molar, soon after which operation the contiguous muscles became painfully sore, while the tooth was apparently well and announced by the dentist as being in no way connected with the inflammation which however increased, finally resulting in the abscess described. Yet the tooth was pronounced innocent of the cause, and retained with the filling in position. The case finally passed into the hands of a physician, who believing that the dentist was correct, spent many months in fruitless efforts to cure. The patient then removing to Philadelphia, placed himself under the care of a physician here, who finally consulted a Philadelphia dentist of deservedly excellent reputation. He also pronounced the tooth free from any connection whatever with the tumor. After spending more time in unavailing treatment, the patient was brought to me for an opinion. Upon careful examination I unhesitatingly expressed a belief that the tooth was the sole cause of the trouble, and was asked by the physician what treatment I would advise, and replied, that as the tooth was dead, isolated, without an antagonizer and positively useless, I would advise its immediate extraction. If otherwise, a removal of the filling and the treatment both through the foramen and the fistula. Whereupon the tooth was removed and in less than ten days the abscess was well, without further treatment, leaving no sign save the scar that it had ever existed. One of the many points of interest attached to this case is its refutation of the frequently expressed belief that no tooth can produce an abscess without becoming very sore, and generally very painful.

Another case very trifling in itself, but showing how much trouble may arise from failure to diagnose correctly, or to seek advice of a specialist to whom its treatment rightfully belongs, was that of a young servant girl brought to me by her mistress after a year's trouble from an abscess immediately under the mesial line of the chin. This patient had been for many months waiting on two eminent surgeons at one of our hospitals. They had already performed two operations, the character of which I of course, know nothing but do know that if no better at her next visit, it was their announced intention to remove the inferior central incisors. At this juncture she was brought to me. It was only after very critical examination that there appeared any indication that their pulps were devitalized. The roots however were covered, for at least the upper third, with a black flinty tartar, which it was very difficult to dislodge. By the time its removal was accomplished, it seemed sufficiently evident that their vitality was extinct; whereupon the chambers were entered through the lingual sulce, the fœtid gases expelled and the roots filled with gutta-percha after three days treatment; a week from which the abscess was entirely cured, leaving nothing but an unornamental cicatrix.

Perhaps the most singular case and its results of an acute alveolar abscess that I ever met with, was that of a young lady, who soon after marriage was brought to me to have her teeth put in perfect order, and in whose mouth was found a devitalized superior incisor. As the tooth was well filled on the approximal surfaces, I entered the pulp chamber through the palatal fissure. The tooth was but slightly discolored, and besides the devitalization seemed to be in an excellent condition, requiring no other treatment than that of purification, which I effected as thoroughly as practicable at one sitting, and temporarily filled with gutta-percha. After which she returned to her home in the country, and I heard nothing of her for a week or ten days. When she did report, it was to the effect that within a day or two after filling, the tooth had become very sore, suppuration took place, from which she had suffered great pain until it broke; after which the tooth remained so loose that it could easily have been extracted with the thumb and finger. I removed the filling, and believing that it would immediately recover, applied a simple dressing, (I think of glycerine,) painted the gums with iodine and requested her to call again in a few days. When she did so, it was found that instead of having improved it was much worse, in every particular, and so loose as to seem likely to fall out, and for the next two weeks, to the best of my memory, would yield to no treatment. As it seemed to be a hopeless case, with ordinary modes of management, I determined, with consent of herself and husband to fathom the mystery by extraction. Upon doing so, the root was found covered to the thickness of the thirty-second of an inch, with a perfectly white fatty-like substance, much resembling minute skippers as though they had been cut in two and fastened by the cut ends to the periosteum and crowded so closely together as to leave no spaces whatever between them; and this condition existed from the extremity of the apex to within a line of the margin of the gum. Having really little hope of finally saving the tooth, I yet thought well to give it a trial, and therefore removed with a dull edged instrument all of the abnormal structure; finding the periosteum in a much better condition than could have been hoped for I thoroughly bathed the root with pure laudanum, served the socket in the same manner, returned the tooth to its place, tied it firmly in situ with floss silk, from which time the disease disappeared; a few months later I filled with gold, having found it sound and apparently firm as ever. It remained perfectly comfortable up to the day of her death, which occurred some two years afterwards of pulmonary consumption.

But it is possible that a devitalized pulp and filled roots may prove dangerous, even in the mouth of a perfectly healthy individual, as will appear from the following circumstance. In the winter of 1862, I think it was, a young college student of most robust health and constitution, came to me to have his teeth, which were in a very bad condition put in order. He had spent most of his vacation in holiday pastime among friends before going to the dentist, leaving but a few days in which to have accomplished work which should have been spread over as many weeks. All then must be done in a hurry; among the many operations needed was the removal of a nerve and filling the root of an inferior bicuspid. The work was accomplished with every possible care and left no thought on my mind that the least trouble was to be apprehended, although everything was necessarily hurried through with the greatest rapidity; the young man immediately upon its completion, hastened to his home in New Jersey, I think, whence in a few days to return to college, but, alas! his college days were ended; from cold or other causes, the tooth became sore, an abscess suddenly formed, and the patient (if the account I afterwards received was correct), actually choked to death from swelling of the neck, the tooth having been allowed to remain in the mouth until very shortly before he breathed his last. A more minute account of the circumstances attending his death, I am unable to render, as what is here stated is all I ever received.

The question will instantly arise upon hearing this recital, as to how such catastrophe could occur if the pulp had been thoroughly removed, and the root perfectly filled. A number of possible conditions might be named, but that one probable in this case may perhaps be best illustrated by the following circumstance.

In the winter of 1856, while practicing in Pottsville, I attended to the teeth of a young man living four miles distant, for whom I removed the pulp and filled the roots of a very large inferior, first molar. The canals seemed to be unusually long, but sufficiently large in proportion, and easy of access to the accomplishment of all the work that seemed necessary with unusual ease, and when completed, I felt as unsuspicious of future trouble as though it had been the most simple operation possible. A few nights later, however, I was awakened by a furious ringing of the door bell. Upon opening the door, which I did myself, there stood my young friend W., for such was his name, having ridden on horseback through a cold driving storm the four miles that lay between his office and mine, with, as the poet hath written: "a raging canal in his mouth." How furiously raging you may suspect from the anxiety manifested to rid himself of it by the four mile ride in the storm. It was of course, extracted as speedily as possible, but after such a tug as dentists rarely meet with; notwithstanding the length of the canal that had been filled, there lay beyond another division and lakelet, in shape like the leaf of the lemon trifolia; as long and as broad as a large grain of wheat flattened, thinner in the centre than laterally, between which and the former there was a contraction to almost nothing. This lakelet was filled with a highly inflamed and congested part of the nerve, which could not have been suspected, and therefore would not have been discovered by any operator.—*Dental Office and Laboratory.*

